# Assessing the reliability of open-source data used for spatial characterisation of urban sanitation infrastructure—a field study in Rajshahi, Bangladesh

**DOI:** 10.1007/s11356-025-36158-0

**Published:** 2025-03-10

**Authors:** M Sufia Sultana, Toby Waine, Niamul Bari, Sean Tyrrel

**Affiliations:** 1 Cranfield Water Science Institute, Cranfield University, Bedfordshire, UK; 2 Cranfield Environment Centre, Cranfield University, Cranfield, Bedfordshire UK; 3 Department of Civil Engineering, Rajshahi University of Engineering & Technology, Rajshahi, Bangladesh

**Keywords:** Urban sanitation, Open-source data, Faecal matter, Fieldwork, Sanitation infrastructure, Rajshahi, Bangladesh

## Abstract

Urban sanitation in rapidly growing secondary cities remains a critical challenge, particularly in unsewered areas. This study evaluates the effectiveness of open-source data for modelling the sources and movement of faecal matter in such contexts, with a specific focus on Rajshahi, an unsewered secondary city in Bangladesh. By triangulating data from direct observations, key informant interviews (KIIs), and focus group discussions (FGDs), we assess the reliability of using open-source data to map faecal matter flow. The findings demonstrate an 80% alignment between the model’s simulated flow directions and actual field observations, highlighting the potential of using open-source data in urban sanitation management. Furthermore, the study reveals that 80% of buildings are connected to storm drains with many allowing faecal matter to overflow from septic tanks, thus posing a health risk. Despite efforts by the City Corporation to manage the drainage system through reactive measures like drain scraping and unblocking, blocked and overflowing drains remain prevalent, particularly during the rainy season. KIIs and (FGDs) highlight a lack of proactive maintenance and widespread unawareness of sanitation management systems. This integration of field-based insights with open-source data supports for a more proactive, data-driven approach to urban sanitation management in secondary cities, ultimately aiming to enhance public health and quality of life.

**Graphical Abstract:**

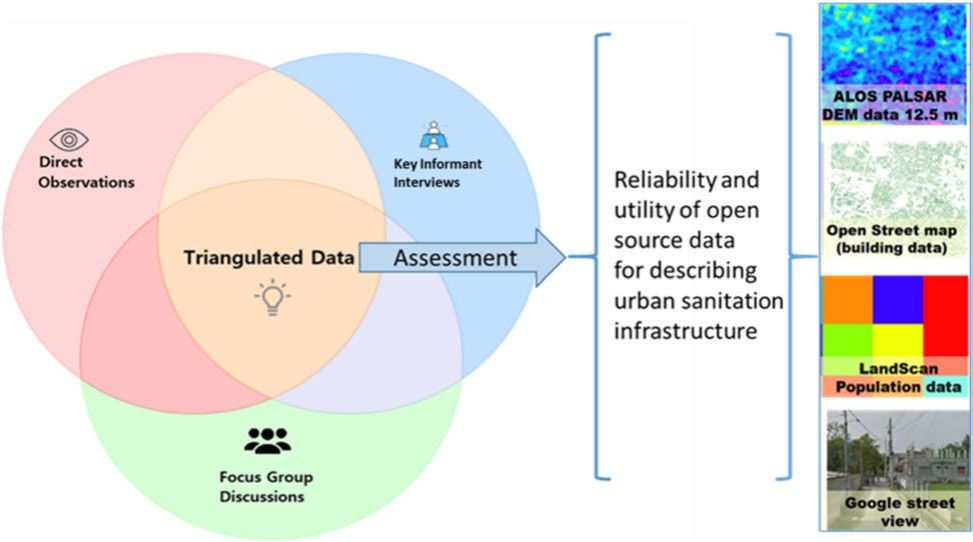

**Supplementary Information:**

The online version contains supplementary material available at 10.1007/s11356-025-36158-0.

## Introduction

Urban sanitation poses significant challenges, often necessitating a spatial perspective because faecal solids, wastewater and urban runoff move in city-scale networks. This need becomes increasingly critical with rising urbanisation and population pressures, demanding a more comprehensive attention to the spatial variability of sanitation systems. The release of faecal matter from septic tanks into storm drainage is a widely known health hazard in urban areas (Ekklesia et al. 2015; Mills et al. 2018; Sercu et al. 2011). Despite the opportunities presented by recent advances in geospatial data and satellite remote sensing, their application in the sanitation sector remains largely underexplored (Leidig et al. 2016). In the past decade, numerous innovative methodologies have been developed to assess faecal matter production and its fate in urban settings (Foster et al. 2021; Peal et al. 2020; Strande et al. 2018). Among these methods, the shit flow diagram (SFD) has proven particularly beneficial, providing an aggregate assessment of the entire city (Peal et al. 2014). However, a more spatially distributed approach was adopted by Sultana et al. (2023) where open-source data has been utilised to spatially represent sanitation systems in a specific area of Rajshahi city, Bangladesh. This novel approach has suggested new insights into faecal production sources and faecal movement pathways at an urban sub-catchment scale. Spatial representation at the building level could lead to significant benefits if city authorities consider applying the model for individual building service delivery. While the spatial scale is smaller, this still has the ability to obtain data that can offer detailed insights into the sanitation situation. Fieldwork and building-level surveys can help us gather information about which buildings have soak pits, use emptying services, or have information about drain scraping.

Regardless of these advances, there is a lack of knowledge about the use of open-source data. Questions about the quality of data and completeness persist, especially regarding its capability to accurately represent the complexities of diverse and individually managed sanitation systems connected to urban drainage networks (Amin et al. 2020). To address these uncertainties, several key assumptions were analysed and five key influencing factors that impact the understanding of spatial patterns of faecal matter movement were identified (Sultana et al 2023, Sultana 2024). These are the emptying of septic tanks, the use of soak pits, sludge removal from drains, variations in faecal matter production by building types, and the presence or absence of toilets. Whilst the research developed several plausible scenarios to assess the impact of influencing factors in the simulation, it was not validated by any field data. Fieldwork is essential to provide additional data, which can be used to refine the model. It is yet to be established whether fieldwork can provide additional clues, which can subsequently be used to improve the methodology. Comprehensive house-to-house surveys, though beneficial, are often unaffordable for municipal authorities. This research explores the use of open-source data supplemented with limited field observation.

This study aims to identify potential enhancements to the methodology for spatial faecal mass flow mapping, assess the validity of assumptions and where possible the model outputs in the test catchment, and deepen our understanding of the sanitation landscape in Rajshahi city. This integrated approach is necessitated by the current low confidence in existing sanitation data, the preliminary nature of a prototype model reliant on limited and untested data, and the imperative to establish the reliability of this innovative mapping method.

## Study area

**Fig. 1:**
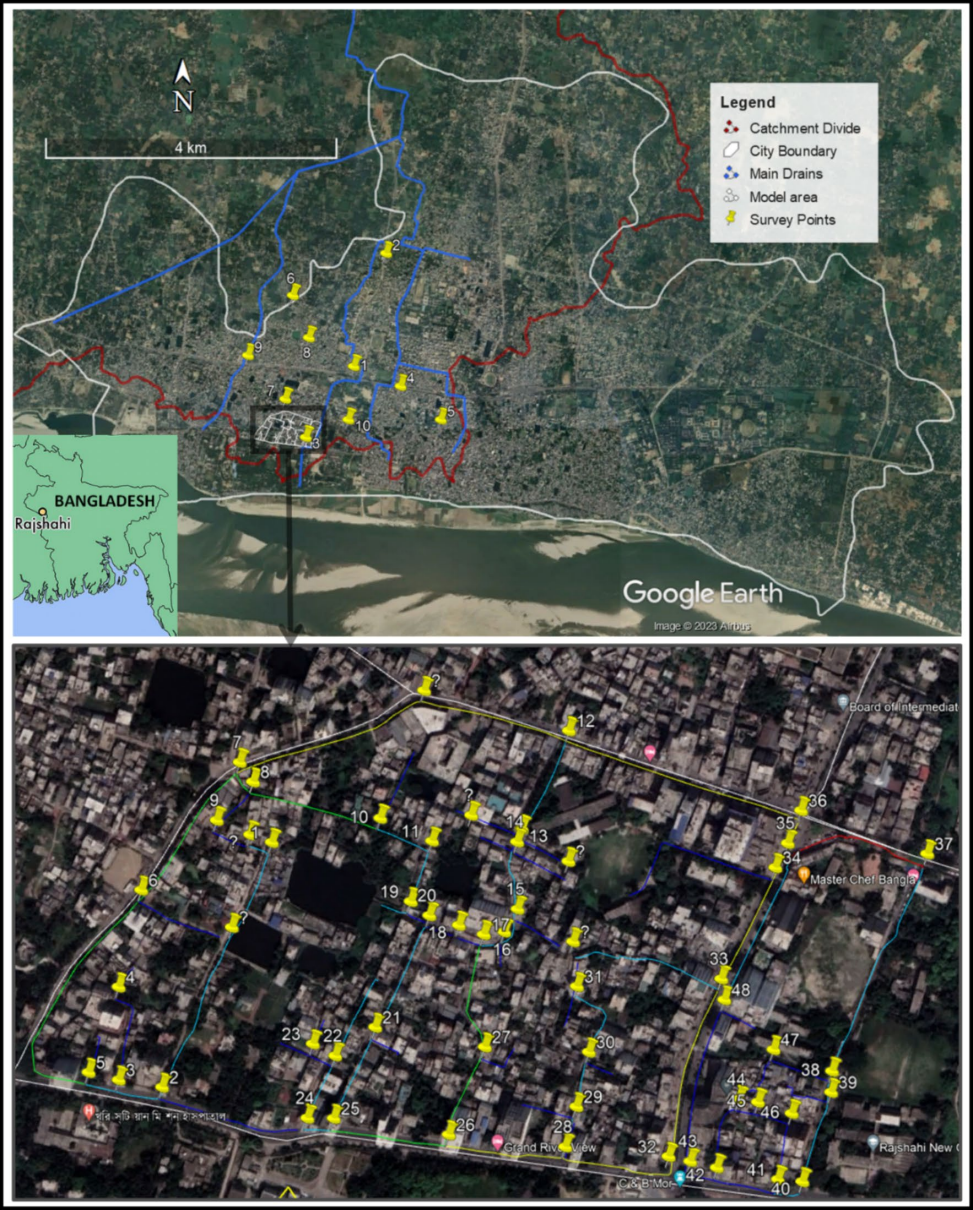
The image of Rajshahi city highlights the study area along with field observation points. The Google Earth image of Rajshahi city at the top shows the city boundary, catchment divide line, main drains of the western catchment, and prototype model area. It also shows the survey location points. The map at the bottom shows the detailed model area and the 48 drain connection points used as direct observation locations

Rajshahi is one of the largest secondary cities in Bangladesh. It is primarily divided into two hydrological catchments. Here, we target the western catchment, drawing on a detailed model from the work of Sultana et al. (2023). This catchment is where topography and hydrological dynamics are intricately related, with surface water flow largely dictated by a south-to-north gradient, directing precipitation and wastewater towards the Baranai River and the Beels to the north of the city (Clemett et al. 2006). The streams, together with the engineered storm drains, form a structured hierarchy that plays a critical role in the area's drainage system. Each major stream, several of which have undergone engineering modifications, creates its own series of sub-catchments. One such sub-catchment, previously modelled by Sultana et al. (2023), is shown Fig. 1.

Sanitation within this catchment is characterised by the absence of a centralised sewerage system, as is the case in other parts of the city, leading to a reliance on individual septic tanks connected to street drains. Septic tanks are typically constructed underground and consist of two chambers, with the first chamber occupying approximately two-thirds of the total tank volume. An inlet pipe directs faecal matter and flushed water into the first chamber, where the separation of liquid and solid waste occurs. The resulting supernatant fluid overflows into the second, smaller chamber and is then discharged into the drainage system or the soak pit through an outlet pipe (Tilley et al. 2014). Pit latrines are generally not used in multi-storey buildings but are more commonly found in slums and temporary housing in underdeveloped areas. Information regarding their specific locations or emptying practices in the city remains limited. Therefore, this study focused on septic tanks and their connections to soak pits or drains. This is complicated by the significant proportion of systems that do not adhere to the national building code (HBRI 2015), which specifies that wastewater from septic tanks should be contained in soak pits rather than connected directly to drains. Many systems instead release wastewater, along with escaped faecal matter, into this drainage system.

According to the drainage master plan of Rajshahi city, there are three types of drains which are classified on the basis of their width. These are identified as primary, secondary, and tertiary drains. Primary drains are the main drain in the area, serving as the principal conduits in the locality, and possess a width exceeding a metre. Secondary drains are medium-type drains connected to the primary drain and tertiary drain, with a width of less than a metre and more than half metre. Tertiary drains have a width of less than half a metre. They are the smallest type of drain, responsible for carrying wastewater away from buildings to secondary drains, which then connect to primary drains (Islam et al. 2021; Tabassum 2020). Some buildings also had direct connections to secondary and primary drains when situated adjacently (Fig. 3).

The area is a mix of residential and commercial zones. Commercial buildings, predominantly small shopping complexes, hospitals, and diagnostic centres, are mainly found in the northern and eastern edges of the model area.

Field observations were primarily conducted within the designated model area, with a few strategically chosen locations outside, within the wider western catchments (Fig. 1). This approach was essential for evaluating and contrasting sanitation infrastructure across broader regions, crucial for enhancing the model for larger catchments or cities. In this study, sanitation infrastructure refers to on-site containment systems, such as septic tanks and soak pits, and associated conveyance networks, including street drains, which manage human waste in the absence of a centralised sewerage system.

## Methods

**Fig. 2:**
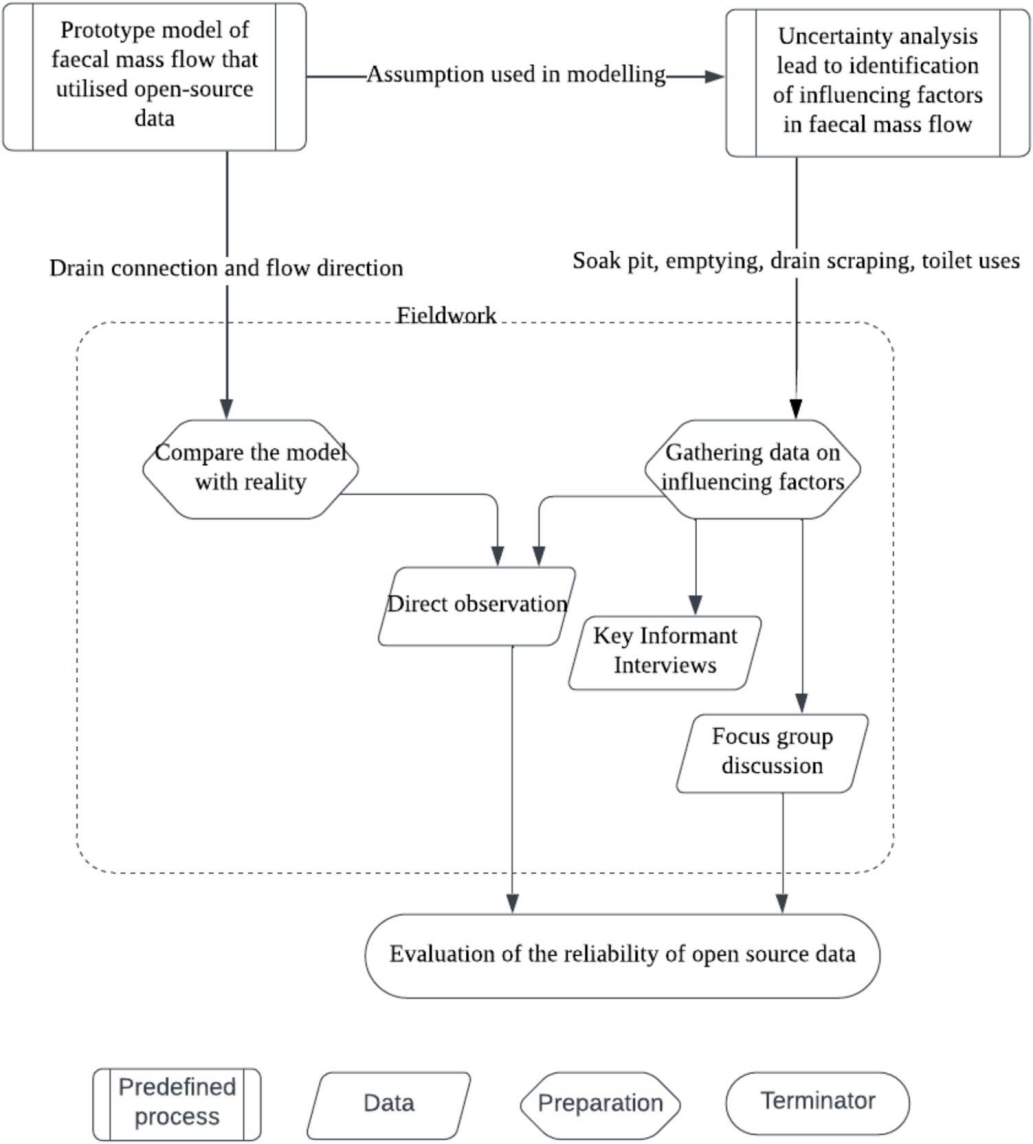
Methodological flowchart outlining the integrated approach of the study, encompassing direct observations, key informant interviews (KIIs), and focus group discussions (FGDs). This chart visualises the sequence and interplay of the methods used. The symbols are conventionally applied: the rectangle represents a predefined process, the parallelogram represents data collection, the hexagon indicates preparation steps, and the oval signifies a terminator or end point

This study employs a wide-ranging methodology to investigate the sanitation infrastructure in Rajshahi, combining various approaches over a 3-week fieldwork period from 3 to 25 August 2022. Figure 2 illustrates this methodology, which includes direct observations, key informant interviews (KIIs), and focus group discussions (FGDs). The weather during this period was mainly dry and humid, with occasional rainy conditions.

The method gathers both quantitative data on sanitation facilities and qualitative insights into faecal management practices. By incorporating observations, the study tests the validity of the open-source data model. Additionally, key informant interviews (KIIs) with city sanitation officials and focus group discussions (FGDs) with local women, who are non-sanitation professionals provide vital context, revealing the factors that influence spatial variability and the movement of faecal matter within the city. The effectiveness and validity of the methodology were systematically evaluated, particularly in terms of its practical application and accuracy when using open-source data for spatial analysis. The following sections provide detailed elaboration on each of these methodological aspects.

### Direct observation

Direct observation was the most important part of this field study to understand the sanitation context and check the reliability of the model. Data collection was carried out using a pre-prepared Google Form with set of questions (Table 1) to check the model’s reliability and understanding sanitation infrastructure and management system that was also, complemented by traditional note-taking. All the responses are made available in the supplementary information.

**Table 1:** Summary of the direct observation questionnaire used to assess model reliability and sanitation infrastructure. The questionnaire was designed to evaluate the model reliability, identify key influencing factors, and gain insights into the sanitation infrastructure. Data collection was conducted at 58 sampling locations

Observation items	Response options	Definition	Rationale
Station ID	Numeric	Unique identification of the location	Provides a spatial reference for data analysis
Latitude and longitude	Numeric	Coordinates marking the precise location	Facilitates spatial analysis and integration with Geographic Information Systems (GIS) tools
Flow direction	North/south/east/west/other	Direction of faecal mass (wastewater) flow in the drain	Validates model-predicted flow directions against observed directions, to assess model accuracy
Drain to drain connection	Yes/no	Whether the drain is connected to the broader drainage network	Indicates potential pathways for faecal mass flow within the drainage system
Width of the drain	Numeric	The measurement of the width of the drains	Assists in understanding the capacity of the drains and identifying potential bottlenecks within the flow system and the reliability of the drainage map itself
Drain cover	Yes/no/partly	Status of the drain cover	Affects accessibility and observation
Visible connection (building and drain)	Yes/no	Whether a connection between buildings and the drainage system is observable	Validates assumptions about the connectivity of drains allowing assessment of model accuracy
Visible faecal matter in the drain	Yes/no	Presence of faecal matter visible in the drain	A visual assessment of the nature of septic tank overflow
Signs of overflow from the drain	Yes/no	Evidence of overflow occurring from the drain	Indicates capacity issues or blockages downstream, relevant for assessing overflow events and their potential for environmental and health impact
Scraped material present	Yes/no	Observation of desludged material on the sides of the drain	Provides insights into maintenance practices and blockage locations, indicating recent cleaning efforts
Water logging	Yes/no	Presence of water in the drain without noticeable flow	Signals areas of stagnant water due to sludge and other solid waste materials, hinting at ineffective drainage and potential health hazards
Complete blockage due to sludge	Yes/no	The drain is completely blocked with sludge and solid waste	Serves as a direct indicator of drain functionality, reflecting severe challenges in sanitation infrastructure
Accumulation of solid waste	Yes/no	The drain is mostly filled with solid waste	Highlights issues with waste management, affecting drainage efficiency and environmental health
Comments	Text entry	Facilitates the capture of qualitative insights and anomalies not directly addressed by the checklist	

One of the main goals was to observe faecal sources and movement pathways through the hierarchical drainage system.

The technique used in this approach was to observe and document the drainage system in relation to the flow direction. To do this, 48 drainage junction points (where two or more drains are joined) were identified, where building-drain connection, flow direction and sign of faecal matter accumulation, as well as a few other sanitation features were checked (Fig. 1). For instance, questions like ‘Is there any visible connection to a building/septic-tank and drain?’ were designed to explore the assumptions used in the modelling (Sultana et al. 2023)—whether all septic tanks are connected to the drains. Observations were made at each location, focusing on buildings adjacent to the sampling points. Multiple buildings were observed at each junction (i.e., sampling point) but were collectively recorded as a single observation. For example, in assessing building-to-drain connections, if three buildings were observed at a sampling point and two of them had visible pipes connecting to the drain, the point was recorded as ‘yes’, indicating that the majority of buildings in that area were connected to the drain. This approach was chosen to simplify the analysis while still capturing representative patterns at each sampling point. While this method assumes that the majority of observed buildings reflect the overall condition of the area, we acknowledge that it may override specific details or outliers, such as cases where a significant proportion of buildings lack such connections. Future studies could explore ways to refine this method, such as recording individual building observations or incorporating weighted analyses, to account for variability more comprehensively. Finally, the percentage of sampling points with a visible building to drain connection was calculated as the number of sampling points logged as ‘yes’ as a percentage of the total sampling point number (*n* = 58).

**Fig. 3:**
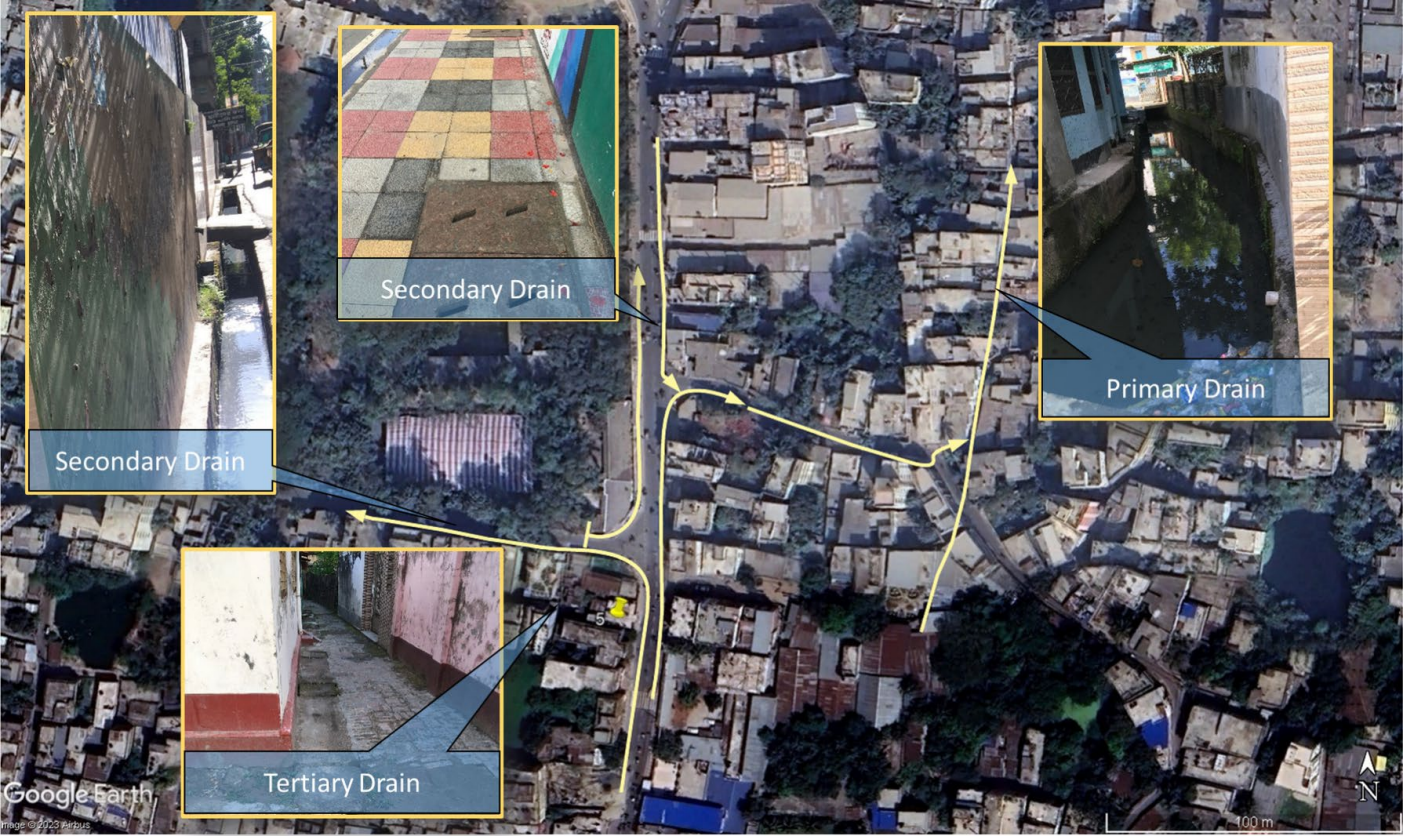
The hierarchy of drainage network and flow direction. The primary drain has a width of more than 1 m within the area which receives the flow from secondary drains (less than a metre in width) which in turn receive flow from tertiary drains of a width of less than half a metre

To assess the consistency of the above findings across larger areas, 10 additional points around the western catchment were identified (Fig. 1). In this case, the sampling points were selected focusing on the primary drains and their connection with other drains (Fig. 3). Similar to the aforementioned sub-catchment level observation, GPS positions were taken at these points, and infrastructure within 100–200 m of each point was investigated instead of investigating few building-to-drain connection, recording similar sanitation aspects as (Table 1).

### Key informant interviews (KIIs)

**Fig. 4:**
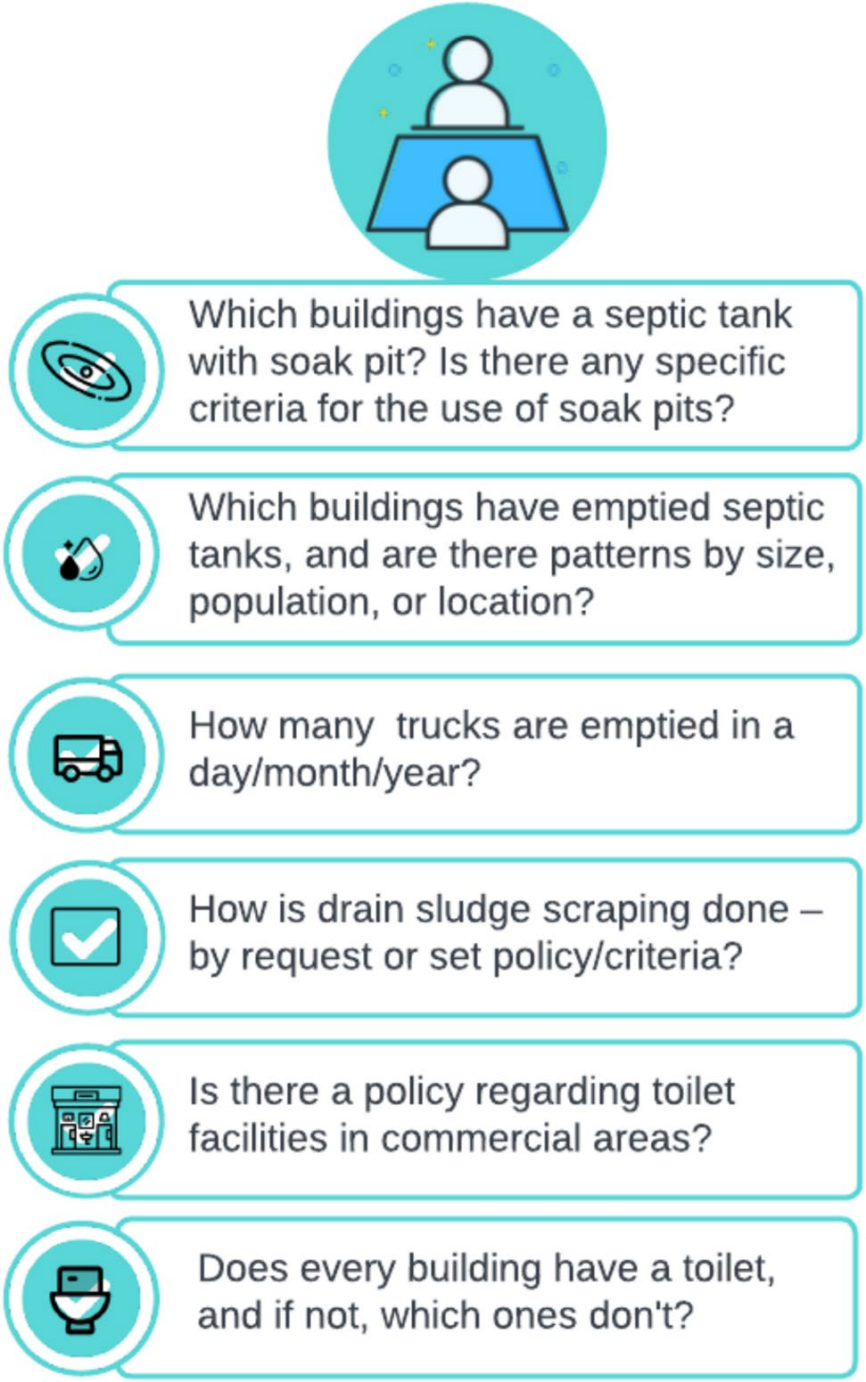
Interview guide for key informant interviews (KIIs) with the sanitation professionals on data availability of influencing factors of faecal matter flow and understanding the sanitation management system of the city

Fieldwork was integral to our study, consisting of direct observations and complemented by purposeful conversations with local residents, as well as structured discussions with sanitation professionals. A broad view of the sanitation landscape was provided by sanitation professionals, identified through our local partners and selected for their direct or indirect roles in the sanitation governance of the city. Consultations with an academic from a local university, three city authority staff members, and four sanitation professionals provided in-depth knowledge on various aspects of sanitation management. Scheduled meetings acted as platforms for sharing research findings and discussing the practical challenges of sanitation management, from septic tank emptying practices to the maintenance of storm drains. To guide these expert discussions, we utilised a interview guide (Fig. 4) that focused on crucial aspects of sanitation management, such as toilet availability, soak pit usage, drain scraping procedures, and commercial toilet policies.

Additionally, informal conversations with local residents occurred during the direct observations. The informal yet targeted conversations revealed critical details about sanitation, such as drain continuity, building drain connectivity, overflow incidents, and cleaning routines. These dialogues not only enhanced our field observations but also were instrumental in creating a nuanced understanding of the sanitation facilities.

### Focus group discussion (FGDs)

**Fig. 5:**
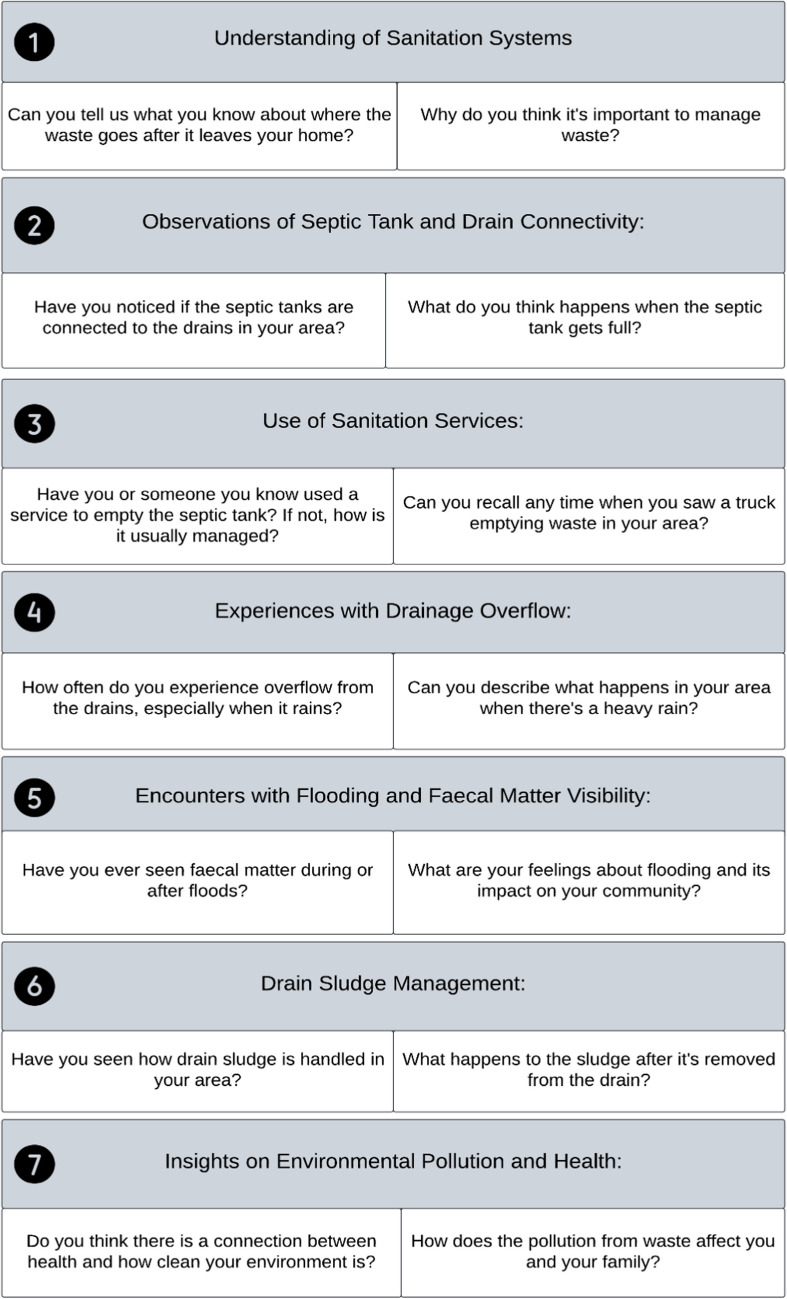
Interview guide list for focus group discussions (FGDs). Topics on the checklist included, building-drain connectivity, incidents of overflow, occurrences of flooding, and cleaning frequency, amongst others

The focus group discussions with residents took place outside the model area to check the sanitation infrastructural similarities within the city. This strategy was crucial for capturing a wide range of viewpoints, essential for understanding diverse experiences and challenges related to sanitation in the city. Including data from various parts of the city ensures a broad and balanced view of the sanitation issues, considering the variety in experiences and perspectives. Extending beyond the realm of KIIs, the implementation of a focus group discussion was undertaken to check the reliability of the assumptions, e.g. septic tank connection to the drain, and to understand the context of sanitation systems from the perspective of the users. A customised interview guide aligning with the aforementioned Interview guide (Fig. 4) was followed here (Fig. 5). The group discussion took place in a densely populated, low-income area of the city.

The focus group discussion was facilitated in collaboration with the local partner. The focus group encompassed twelve local women, who are the members of The Livelihoods Improvement of the Urban Poor (LIUPC) Project, from low-income families. Prior to the discussion, the researcher obtained the participants' consent, clarified the research goals, and ensured understanding of the subsequent use of the data collected. A checklist (Fig. 5) was used to guide the discussions, ensuring the collection of information and enabling participants to share their experiences and insights on the topic. Topics on the checklist included, building-drain connectivity, incidents of overflow, occurrences of flooding, and cleaning frequency. The participants were encouraged to share their experiences and knowledge on various topics, including environmental pollution.

## Results and interpretation

The outcomes of this study, presented here, are designed to meet the research aim: assessing the validity of model assumptions and outputs, and improving the methodology for spatial faecal matter flow mapping and enhancing the understanding sanitation context of Rajshahi city. These findings are categorised into direct observations, informal interviews, and focus group discussions. The integrated findings from the field investigation are presented in Table 2 and described in relation to the aim of the study in the discussion section. The direct observations substantiate parameters of the model through physical inspections and local interactions in the prototype area and wider catchments. The KIIs and FGDs further evaluate the insight of sanitation management practices and data availability of influencing factors of faecal matter flow, and understanding of the sanitation system of Rajshahi.

**Table 2:** Summary of the results of the direct observations, informal Interviews, and focus group discussions and indicating the extent of confirmation of the assumptions

Assumptions	Observation	Data source	Level of confirmation	Implications for the prototype methodology
All septic tanks are connected to the drains rather than a soak pit	Around 80% of buildings are connected to drains, according to direct observation and survey data. Informal interviews and focus group discussions confirmed this widespread practice, although it is not the official expectation. Some soak pits are also connected to drains to prevent overflow, particularly during the rainy season	Direct observations, informal interviews, focus group discussions	Medium/high	The prototype methodology will give precedence to drain connectivity in its spatial representation. There is a need to determine representative criteria for buildings linked to soak pits rather than drains, to exclude the contribution from the drain
Septic tanks connected to drains are not emptied	An informal talk with a member of the sludge emptying management team and a focus group discussion indicated that septic tank emptying service via the emptying track is not widely used Also, the use of manual emptying systems is prevalent, where sludge is often dumped into the nearest primary drain	Direct observations, informal interviews, focus group discussions	Medium	The methodology should consider local septic tank emptying practices, particularly manual emptying. Also, identifying map able criteria for buildings undergoing emptying is vital to exclude their drain contributions
All of the sludge entering the drains from buildings is transported to the catchment outlet	Sludge entering to the drain was evidenced by the presence of sludge in drains, blockages, scraped sludge by the side of the drain and post-flood mud layers on roads. But all the sludge transported to the outlet was not true because the scraped sludge transported to the informal dumping side, outskirt of the city	Direct observations, informal interviews, focus group discussions	Medium	The method should account for faecal accumulation in drains, excluding the amount of sludge scraped off
Faecal matter is produced at all buildings irrespective of their types	Commercial areas, particularly those with dual-purpose buildings (residential and commercial), likely produce more faecal matter than that assumed in the model due to the influx of the daytime population. All commercial floors observed contained toilet facilities	Direct observations	Medium	The methodology should distinguish between building types and potentially assess population influx in commercial zones to measure faecal matter production reliably
Every building has a toilet and septic tank	Majority of the buildings use septic tanks, although the city authorities do not have exact data on this. Informal interviews revealed the widespread presence of septic tanks, including the older buildings built before the 1980s. However, the septic tank's design and connection to the drain may differ	Informal interviews	Medium/low	The methodology should consider adjusting for variability in containment designs. Specifically, differentiation might be needed for buildings lacking toilets, such as warehouses

### Direct observation

**Fig. 6:**
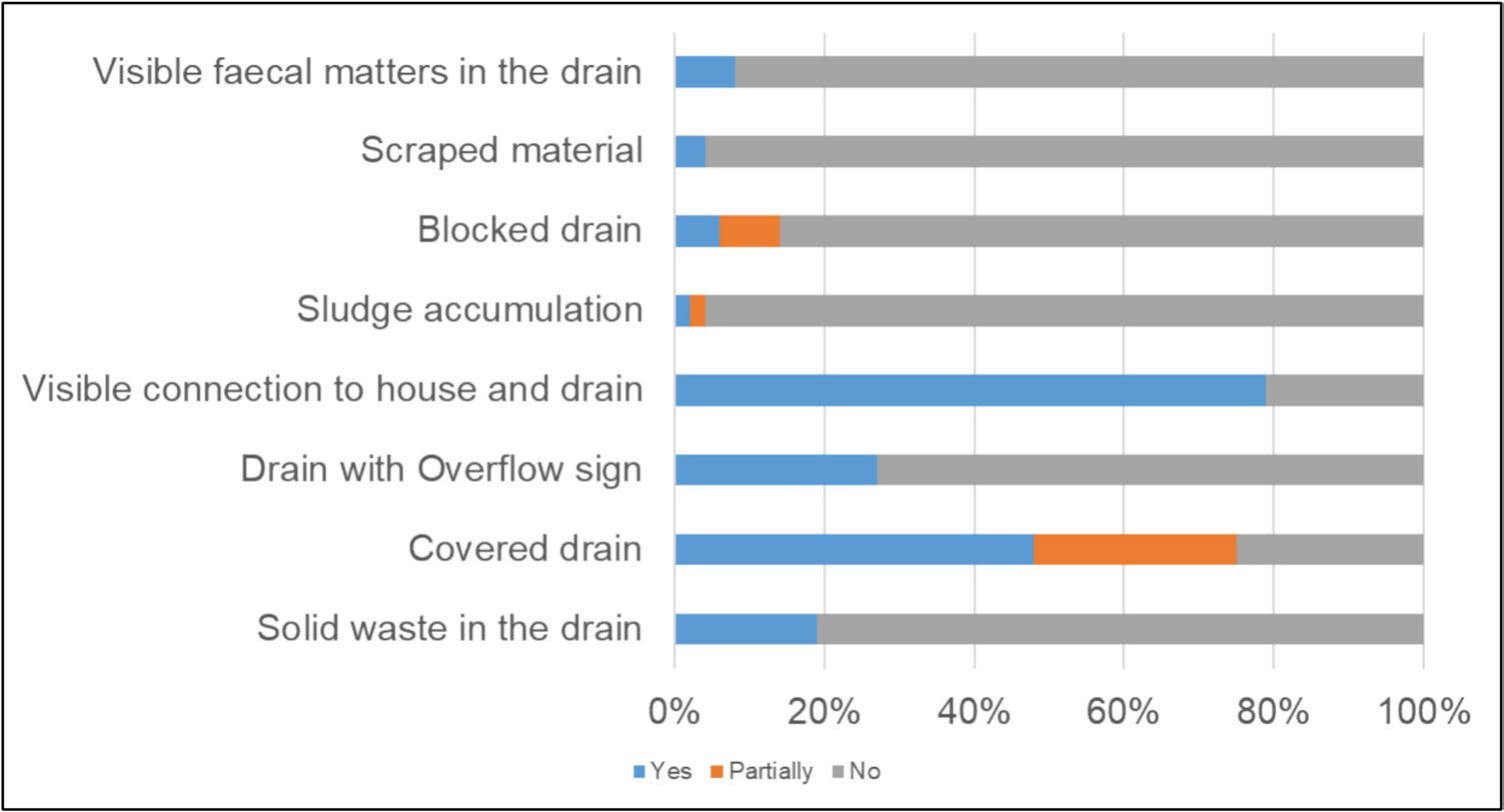
Summary of direct field observations. The total number of sampling point was (*N* = 58), 10 of which were conducted outside the prototype model area in wider Western catchment

In the city, the majority of the buildings use septic tanks and these are connected to the drain for the wastewater management. The survey result (Fig. 6) showed that around 80% buildings are connected to drains based on 58 sampling location. It is also observed that while most drains are covered, primary drains are usually not. The results showed that around 50% of the drains were completely covered with concrete slabs, with the rest being either partially covered with the same material or not covered at all. All drains, covered or uncovered, are hydraulically open channel, i.e. flow is gravity driven (Li et al. 2020). Flow directions and water in these drains can be seen directly even if they are covered since they have manholes for maintenance and the manhole lid has multiple small lock holes, large enough, to show the water within and its flow. It was also observed that some drains, mostly secondary ones, backed up when it rained, while some (~ 5%) tertiary drains were found to be blocked with the solid waste including metal cans, plastic bags, paper carton box and sludge-like sediment of uncertain composition or origin.

In here (Fig. 6), the bar plot illustrates eight evaluated sanitation attributes in Rajshahi city: visible faecal matter, scraped material presence by the side of the drain, blocked drains, sludge accumulation in the drain, visible building-to-drain connection, signs of overflow, covered drains, and solid waste in drains. The results, converted to percentages, indicate each attribute’s presence (Yes), partial presence (Partial), or absence (No). Notably, scraped material presence, blocked drains, and sludge accumulation in the drain were considered indicators of faecal presence, alongside visible faecal matter, cumulatively accounting for 30% of the sampling points. The study also reveals that approximately 80% of buildings have visible connection to a drain. The majority of drains (75%) are either fully or partially covered. Other observations include the presence of solid waste in drains at around 20% of locations and signs of overflow in approximately 25% of the locations.

A number of indicators are used to identify the presence of faecal matter in the drain because the ‘sludge’—a semi-solid mixture of various waste materials, including organic waste, sediment, debris, and escaped faecal matter—forms a dense, viscous substance. The presence of faecal matter accelerates this accumulation due to its organic nature facilitating sediment aggregation (flocculation), making sludge an important indicator for clogging the open drains (Deng et al. 2022; Niwagaba et al. 2014). The indicators used may not be solely associated with faecal matter as other solid waste and road wash materials also gets trapped in the sludge. In approximately 30% of the observation points, these signs indicated the presence of faecal matter in the drain.

Drain scraping is a critical activity within the city’s sanitation management system. The procedure is essential for preventing the overflow and to maintain a functioning drainage system for environmental health and urban well-being. The city corporation has a work force to manage and maintain the drain scraping regularly. Scraped sludge typically remains on the roadside for hours or even days to allow the liquid part to run off back to the drain, making it easier for city cleaners to transport the semi-dried material. During the fieldwork, there were relatively few drains with scraped-out sludge. Field observations highlighted the practicalities of this approach. For instance, during a specific operation at observation point 9 within the prototype area, sludge was left on the roadside overnight. By the subsequent day, it had been removed. This quick removal was the part of service improvement which helps to reduce potential health risks to the public.

A significant expansion of flooding throughout the city during the rainy season, particularly at junctions where numerous secondary drains converge before joining the primary drain was noted. The sludge accumulation most likely influences the flooding as it reduces the flow capacity. Within our model area, the northeast corner was especially susceptible to flooding. Moreover, flooding also occurred in the northwest corner of the model area, mainly due to water inflow from a nearby sub-catchment. This floodwater dispersed drain sludge widely, and as the floodwater receded, we discovered a layer of residue on the roads and dry roadside areas. These observations offer crucial insights into the dynamics of urban flooding and its role in spreading faecal-contaminated sludge throughout urban areas.

Commercial areas, particularly on the model area’s eastern side, are concentrated with health care facilities, and the location is close to the largest government medical college hospital. These areas, which extend to the north-eastern boundary, draw a significant number of people, thus augmenting the daytime population. Notably, many commercial buildings in these areas have a residential function as well, with residences typically situated on the upper floors. All the commercial floors contain toilet facilities. Although faecal production in commercial and residential buildings might be similar, the influx of daytime population in these dual-purpose buildings could potentially increase their contributions. This is a critical factor to consider in designing sanitation management systems.

**Fig. 7:**
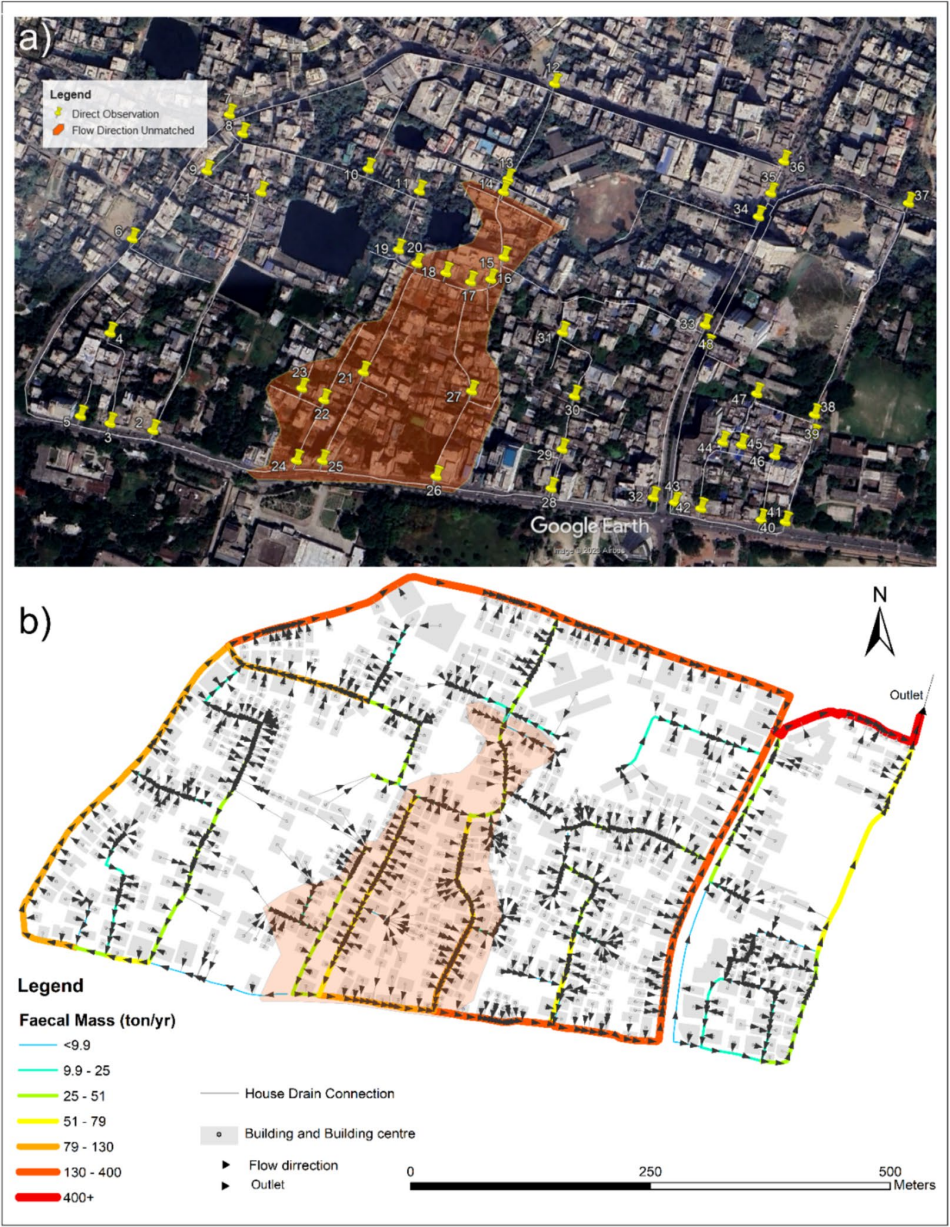
Comparison of modelled flow directions with field observations, highlighting areas of agreement and discrepancies. **a** Map of the 48 drain connection points directly observed to compare the prototype model with field reality. Flow direction discrepancies are highlighted in the coloured areas, where storm drains are engineered to redirect water against the natural slope to mitigate overflow risks. **b** Spatial distribution of faecal mass flow, house-drain connections, and modelled flow directions. This panel illustrates areas of alignment and discrepancies between the model and field observations, emphasising regions where flow patterns deviate due to engineered adjustments

The comparison between the model output and field observations demonstrated an 80% consistency in the predicted flow direction (Fig. 7). The figure illustrates the spatial distribution of faecal mass flow pathways, house-drain connections, and modelled flow directions, alongside the locations of field observations. Areas of agreement between the model and field data are evident, while discrepancies are observed in regions where the engineered drain gradients oppose the natural land slope.

In the highlighted part of the figure, the observation points recorded flow patterns that diverged from the modelled flow directions, reflecting these engineered adjustments. Here, the modelled flow direction was generally southward, followed by eastward flow, whereas in reality, the flow direction was engineered northward before turning east. The spatial distribution of faecal mass flow and the alignment of observed and modelled flow patterns are visualised in the figure, highlighting both the consistency and the localised discrepancies identified during the field survey.

### Key informant interviews (KIIs)

Meetings with local government specialists revealed that there was no formal system in place for collecting sanitation service delivery statistics, such as how many buildings empty their septic tanks yearly/monthly. Nobody knew the exact number of buildings connected to the drainage system. Most of the buildings use septic tanks, some of which are connected to soak pits while others are connected to drains. Formally, buildings are not supposed to use septic tanks directly connected to the drain; instead, they are expected to use soak pits (HBRI 2015). However, it was brought up during the conversation that the practice of house drain connection is widespread because there is no formal monitoring and enforcement. These connections often create almost direct linkages from the septic tanks to the drains, suggesting an absence of soak pits, particularly if the building is very old. This was affirmed through discussions with drainage workers. It was further noted, from these conversations, that some soak pits were connected to the drains to mitigate overflow. This is due to a rise in water table during the rainy season, causing soak pits to be ineffective. Moreover, soak pit designs varied across individual buildings. While some maintained grain-size stratifications in their filter design, others discreetly included an overflow connection with the drain.

In each ward of the city, the city corporation employs a team to manage the drainage systems through sludge scraping and cleaning the blockage. Contrary to the prototype model’s assumption that all generated faecal matter reaches the outlet, the team cleans drains as on-demand services when blockages occur. However, this service is not immediate or entirely efficient due to potential upstream sludge accumulation that can cause recurring blockages. This insight came from the unstructured conversation with local residents.

Unstructured interview with a cleaning team in ward 20, located on the eastern border of the western catchment, provided valuable insight into their operations. The team of 17 includes 10 members scraping drains and seven transporting sludge in rickshaws, which are designed as leak-proof but sometimes leak, to informal dumping sites on the outskirts of the city. They affirmed that recent instructions from the city authority discouraged leaving sludge on the roads to minimise exposure hazards. On a daily basis, they manage to clean about 8 to 10 m (25 to 30 feet) of drain length and transport 3 to 4 van-loads, each carrying 100 kg, of sludge. The vans, locally made rickshaws equipped with containers for carrying wet sludge, are intended to be leak-proof, though they often fail to meet this standard.

Because there is no recorded data available, estimating the volume of sludge removed from the drains presents a significant challenge. Consequently, any calculation or model developed under this assumption does not fully capture the actual dynamics of sludge removal. Therefore, it is essential to approach such estimates with caution, acknowledging the potential discrepancies between assumed and actual sludge movement within the drainage system. The city has mainly two types of emptying system.

The city corporation has an emptying truck which empties septic tanks on demand and takes sludge to the informal dumping site located on the outskirt of the city in the northwest corner. The city authority does not maintain records on the number of truck trips made, but they do keep some form of records regarding truck assignments, primarily for the purpose of fuel billing. Another type is manual emptying system, this is also on demand service and individually managed. When a homeowner calls the cleaner, who is typically self-employed, they arrive and empty the septic tank. They frequently dump the sludge into the nearest primary drain since it is more convenient and less expensive to do so because the dumping site is outside the city. This clearly poses a health risk.

### Focus group discussion (FGDs)

Participants in the focus group discussion expressed limited familiarity with formal sanitation management systems, particularly regarding how faecal management operates or its broader significance.

Participants described frequent drainage overflows, particularly during the rainy season, linking these incidents to heavy rainfall and flooding. They reported that faecal matter is sometimes visible during these events and that post-flood conditions often leave sludge deposits on roads, resulting in layers of dark grey muddy residue.

Regarding drain scraping, participants noted that this activity occurs in their areas. They explained that sludge is typically left overnight to allow liquids to drain before removal by the city’s cleaning team. When asked whether the time spent leaving slurry sludge behind had decreased, participants agreed, echoing findings from unstructured interviews in the prototype model area.

However, regarding the emptying, they are not familiar with utilising emptying trucks for septic tank servicing, some of them thinks the tanks are connected to drains also; manual emptying practices prevalent in their community.

While participants indicated limited familiarity with formal sanitation management processes, their detailed accounts of local practices and issues suggest a practical awareness of sanitation challenges in their area. These insights offered valuable confirmations of the direct observations, contributing meaningfully to the research objectives.

## Discussion

### The model vs reality: fieldwork findings

The fieldwork findings not only support the merits of open-source data but also showcase how the prototype methodology can be paired with traditional methods to bring about a spatial representation with reduced uncertainty. However, the assumption that all buildings connect to the drainage system may not be the reality. This study, through the fieldwork, confirmed that open-source data, while useful, could not provide all necessary details such as which buildings have soak pits, use emptying services, or information about drain scraping (Table 2).

The triangulation of methods—field observations, informal interviews, and focus group discussions—strengthens the validity and reliability of the model’s findings (Patton 1999). For instance, open-source data enables the modelling of spatial patterns of faecal matter generation and movement, while field observations provide essential information to improve the model's reliability. Combining qualitative and quantitative approaches enhances the research, providing a well-rounded perspective (Table 2). This observation was validated by unstructured interviews and focus group discussions, highlighting the absence of a clear pattern of the buildings with septic tanks or soak pits connected to drains. This lack of identifiable trends highlights the need for fieldwork and building-level surveys. However, the field study has emphasised the potential benefits of combining open-source data with monitoring and operation data.

### Challenges and areas of methodology improvement in spatial representation

Sanitation management often takes a back seat in city governance, generally addressed as part of other management activities (e.g. storm drainage maintenance). In cities like Rajshahi, this is because the drains were never designed to be a part of a city-wide sanitation network. Consequently, no proactive framework exists for managing, maintaining, or monitoring sanitation processes. The scarcity of sanitation data, which was observed during the informal interviews, stresses this fact. The sub-catchment prototype method can be employed using only open-source data, providing preliminary spatial insights. However, its effectiveness and accuracy would be significantly enhanced if supplemented with data from municipal sanitation professionals. If feasible, conducting targeted fieldwork will further enrich the outcomes (Fig. 6).

Yet, the validation process for representing buildings in cities is riddled with challenges and uncertainties. For instance, each house could have multiple connections to the drainage system (including grey and black water pipes), some of which may not be visible, but nonetheless exist (Bari 2017). Informal discussion revealed that pipe connections are often installed beneath the drain’s lowest water level, making them invisible.

Identifying faecal matter in the drain is challenging because the moisture content of fresh faecal matter can range from 60 to 85% (Woolley et al. 2014). On top, the user uses toilet flush which causes faecal matter to be mixed and diluted with water. Furthermore, a person uses another ~ 100 L of water on a daily basis which also ends up in the drains where escaped faecal matter from the septic tank gets diluted again. Given that dilution is an ongoing process and repeats downstream of each building, the ratio of grey water to escaped faecal matter is extremely high, making it difficult to distinguish faeces from water.

Moreover, city authorities typically provide on-demand sanitation services, responding to residents’ requests for assistance. However, they often do not maintain data about these service calls, and they may not always respond to all requests. Their records tend to focus on service deliveries for specific activities, such as the addresses requiring truck dispatches. Although not originally intended for sanitation improvement, this data could potentially be leveraged for sanitation management purposes. The service book contains the street name (not which building) where emptying service happened, this information can be incorporated in future in the spatial analysis.

Gathering field data, recording and maintaining databases, as well as conducting data analysis and reporting, are cost-intensive processes, often leading to their limited implementation. However, the integration of open-source data with these activities could significantly enhance the accuracy of the model. This combination would provide a more precise depiction of real-world situations, thereby improving the model's precision and effectiveness.

The method addresses the intersection of sanitation management and open-source data, filling gaps in our current understanding and contributing to more effective and reliable utilisation of these emerging resources for sanitation management. This spatial model can enable city authorities to proactively identify buildings or streets with higher faecal accumulation that require attention before problems escalate, potentially preventing issues that could affect the entire downstream catchment. While residents may report blockages, relying solely on reactive measures can lead to delays, as blockages may remain unreported or worsen over time. By using this model, authorities can implement preventive maintenance strategies, optimise resource allocation, and reduce the overall risk of disruptions to the sanitation system, particularly in high-risk or underserved areas.

While storm drainage systems are not typically intended to serve as faecal management systems, practical realities often necessitate such repurposing in many cities lacking centralised or decentralised faecal management systems. Thus, the modelling using open-source data proposed by Sultana et al. (2023) could be enriched with field observations and further informed by monitoring and operational data. Currently, the main challenges include the exposure to faecal contamination due to flooding and desludging of drains, and the nuisance caused by odour. To mitigate these risks, an interim solution could be implemented for sanitation management. This would involve using the model to understand the spatial dynamics of faecal generation and movement, allowing city authorities to prioritise their efforts to desludging the drain and the maintenance more effectively. Monitoring and operational data, such as locations and frequencies of emptying or scraping activities, could serve as valuable inputs. For instance, in a city, faecal sludge that escapes the containment can increase the accumulation rate in the storm drain and may not reach the outlet as it could settle in the drain first (Deng et al. 2022; Niwagaba et al. 2014). Therefore, managing the sludge through regular scraping, and identifying the underlying causes with the help of operational data, can contribute to improved sludge management. This not only aids in reducing the risk of flooding but also ensures the free flow within the drains, all while proper containment and treatment facilities are being established.

### Contextual implications of spatial representation

Sanitation management operates within a complex, multi-scale environment (Hyun et al. 2019; Scott et al. 2015). The prototype model, which represents a small hydraulic sub-catchment—a unit within a larger catchment where both rainwater and wastewater meet into drains that eventually lead to the catchment’s outlet. These sub-catchments, adhering to a hydraulic hierarchy, combine to form a larger catchment (Li et al. 2020).

City authorities often partition the city into multiple wards for administrative purposes and service delivery. Despite being predominantly used for administrative activities like storm drainage and sanitation, the natural hydrology of many cities often aligns these wards with hydrological sub-catchments. As confirmed through informal dialogues with sanitation professionals and focus group discussions, the city is divided into 30 wards, each with its own reactive management system. The alignment of administrative boundaries with hydrology provides an opportunity to use the spatial representation approach for planning purposes.

One challenge identified with the prototype model is the lack of granular data at the building level, especially regarding factors influencing faecal matter movement. These are the emptying of septic tanks, the use of soak pits, and sludge removal from drains, variations in faecal matter production by building types, and the presence or absence of toilets. While this precise data may not be readily available, city authorities might be able to obtain reliable statistics at the ward level, shedding light on practices like, emptying services, and drain scraping. For example, the city council could track the number of sludge emptying trucks servicing a particular ward over specific periods, such as monthly or annually. Representing this data at the ward level allows a more sophisticated understanding of sanitation management, essentially adding another layer to the current model. This enhanced perspective would serve as a crucial resource for informed planning, strategic development, and the efficient allocation of resources. Also, by incorporating actual population data instead of estimated figures from LandScan, the up-scaled model could enhance accuracy and confidence in sanitation management at the ward level.

Although the need for systematic data collection is well recognised in developed countries, this study highlights its critical importance in data-poor contexts like Bangladesh, where such practices are underdeveloped. Combining these improvements with spatial models and fieldwork could significantly enhance sanitation management in secondary cities.

## Conclusions

This study has demonstrated the effectiveness of using open-source data to model the sources and movement of faecal matter in unsewered urban areas, focusing on Rajshahi, a secondary city in Bangladesh. By integrating data from direct observations, key informant interviews, and focus group discussions, the research assessed the reliability of open-source data for mapping faecal matter flow and identified key challenges and opportunities for improving urban sanitation management.

The findings reveal that open-source data can be valuable for understanding the spatial variability of faecal matter flow in unsewered urban areas. A notable 80% consistency was observed between modelled outputs and field observations regarding flow directions and drainage connections, suggesting that open-source data can offer a reasonable approximation of faecal matter dynamics where detailed local data is lacking. However, while open-source data provides an initial understanding, it is limited by the inherent uncertainties of remotely sensed data, particularly in capturing specific practices such as septic tank emptying, soak pit use, and drain maintenance. These limitations highlight the need for fieldwork to validate and enhance the data’s accuracy, as remote sensing alone cannot effectively monitor issues like blocked or overflowing drains.

While field observations are crucial for supplementing the data, they alone are not sufficient. The study highlights that certain information, such as the frequency of septic tank emptying or the use of particular sanitation facilities, is often not systematically recorded or readily observable. Therefore, collaboration with local authorities is essential to access administrative data that can enhance the model’s precision and applicability to local conditions. This partnership would enable a more comprehensive understanding of the sanitation landscape, bridging gaps that neither open-source data nor fieldwork can fully address on their own.

Looking forward, improved service delivery records and data collection by city authorities could significantly enhance the use of open-source data models for proactive sanitation management. Better record-keeping and systematic data collection at the local level would refine the model and make it more adaptable to the city's operational framework, ultimately supporting more effective sanitation planning and management efforts.

## Supplementary Information

Below is the link to the electronic supplementary material.

Supplementary file1 (CSV 5 KB)

## Data Availability

The data from field observations is included in the supplementary information.
